# Hybrid Whole Genomes of *Brucella melitensis* from Tunisian Animal Isolates: Virulence Factors, Antimicrobial Susceptibility, and Phylogeny

**DOI:** 10.3390/microorganisms13071651

**Published:** 2025-07-12

**Authors:** Ibtihel Ben Abdallah, Germán Kopprio, Awatef Béjaoui, Susanne Köhler, Kaouther Guesmi, Sana Kalthoum, Jacob Gatz, Amel Arfaoui, Monia Lachtar, Haikel Hajlaoui, Mohamed Naceur Baccar, Holger Scholz, Abderrazak Maaroufi

**Affiliations:** 1 Group of Bacteriology and Biotechnology Development, Laboratory of Epidemiology and Veterinary Microbiology, Institut Pasteur de Tunis, University of Tunis El Manar (UTM), Tunis 1002, Tunisia; awatef.bejaoui@pasteur.tn (A.B.); amelarfaoui02@gmail.com (A.A.); abderrazak.maaroufi@pasteur.tn (A.M.); 2 Centre for Biological Threats and Special Pathogens, Highly Pathogenic Microorganisms (ZBS 2), Robert Koch-Institute, 13353 Berlin, Germany; koppriog@rki.de (G.K.); koehlers@rki.de (S.K.); gatzj@rki.de (J.G.); scholzh@rki.de (H.S.); 3 Centre National de Veille Zoosanitaire, Ministry of Agriculture, Water Resources and Fisheries, 38 Avenue Charles Nicolle, Tunis 1082, Tunisia; guesmikaouther09@yahoo.fr (K.G.); kalthoum802008@yahoo.fr (S.K.); lachtarmonia@ymail.com (M.L.); haikelhajlaoui@gmail.com (H.H.); baccar.vet@gmail.com (M.N.B.)

**Keywords:** aborted sheep, whole-genome sequencing, cgMLST, brucellosis, antibiotic resistance

## Abstract

Brucellosis remains endemic in Tunisia, causing abortions in small ruminants, and represents a public health threat through occupational exposure and the consumption of contaminated animal products. The aims of this study are to assess the antibiotic susceptibility of two *Brucella melitensis* isolates (TATA and SBZ) from aborted sheep, to analyze their genomes using hybrid whole-genome sequencing, and to investigate their antimicrobial resistance (AMR), potential virulence factors (VFs), and phylogenetic relationships. Both isolates were phenotypically confirmed to be susceptible to doxycycline, gentamicin, rifampicin, streptomycin, and trimethoprim–sulfamethoxazole, and no corresponding classical AMR genes were identified. However, several potential AMR-related genes (*mprF*, *bepCDEFG*, *qacG*, and *adeF*) and a mutation in the *parC* gene were detected. The analysis of the genotypes revealed 74 potential virulence genes, primarily involved in lipopolysaccharide synthesis and type IV secretion systems. Genomic comparison showed over 99% nucleotide identity between the Tunisian strains, *B. melitensis* bv. 1 16M and *B. melitensis* bv. 3 Ether. Five gene clusters, including three hypothetical proteins with 100% identity, were detected exclusively in the TATA and SBZ strains. Additionally, two unique gene clusters were identified in SBZ: a rhodocoxin reductase and another hypothetical protein. Both isolates were assigned to sequence types ST11 and ST89. Core-genome-based phylogenetic analysis clustered both strains with biovar 3 and ordered the Tunisian strains into two distinct groups: TATA within Tunisian Cluster 1 is closely related to strains from Egypt and Italy, while SBZ near MST Cluster 4 is more related to isolates from Austria and two outliers from Italy and Tunisia. This study provides the first genomic characterization of *B. melitensis* from aborted sheep in Tunisia and offers valuable insights into AMR, virulence, and phylogenetic distribution.

## 1. Introduction

Brucellosis is one of the most important zoonotic infectious diseases worldwide, caused by several species of the genus *Brucella*. Fourteen species are currently identified based on shared biochemical traits and host preferences [1]. *Brucella melitensis*, *Brucella abortus*, and *Brucella suis* are the most virulent species impacting human health and livestock, causing significant economic losses in the livestock sector [2]. These losses result primarily from abortion and infertility in females leading to decreased milk and meat production [3,4,5,6]. In humans, the mentioned species with the exception of *B. suis* biovar 2 cause a debilitating flu-like illness, which can lead to severe chronic complications if left untreated [6,7]. Human brucellosis presents a broad clinical spectrum of symptoms including undulating fever, excessive sweating, malaise, arthralgia, myalgia, loss of appetite, weight loss, hepatosplenomegaly, spondylitis, and arthritis [8].

*Brucella melitensis* biovars (1, 2, and 3) are the main causative agents of caprine and ovine brucellosis, though some cases have been reported in cattle, buffalo, yaks, dogs, and camels [1,9]. The transmission of *B. melitensis* primarily occurs due to exposure to the placenta, fetal fluids, and vaginal secretions expelled by infected ewes and goats during abortion or parturition. Moreover, these fluids can remain infectious in the environment for several months, extending the risk of disease transmission [9]. Brucellosis in livestock affects primarily the reproductive system and presents a wide variety of clinical signs, many of which may remain asymptomatic. In small ruminants, infection with *B. melitensis* is predominantly characterized by abortion in the last third of pregnancy, arthritis, retained fetal membranes, weak offspring, orchitis, epididymitis, and infertility [10,11]. Infected animals and their products are the primary source of brucellosis in humans. The consumption of unpasteurized milk and dairy products or direct contact with infected animals is the predominant cause of *B. melitensis* infection in humans [12,13], with approximately 500,000 new cases annually [12,13]. In many developed countries, brucellosis has been effectively controlled through rigorous prevention and eradication programs. However, it remains endemic in numerous low- and middle-income countries. This is particularly evident in regions where goats and sheep serve as primary livestock and vital sources of economic livelihood, such as the Mediterranean basin, the Middle East, Central Asia, Sub-Saharan Africa, and parts of Latin America [11].

In Tunisia, the breeding of ruminants plays a vital role in national economy; nevertheless, this sector is still impacted by brucellosis, a notifiable disease under Tunisian regulations [14,15,16]. Despite ongoing surveillance and vaccination efforts, brucellosis remains endemic in Tunisia and threatens public health [17,18]. *Brucella* infections have been reported in livestock farms, food products, and humans, yet no genomic research has been conducted on Tunisian animal strains. A retrospective study reported that the prevalence of *Brucella* infection in ruminant herds across Tunisia varied from 0% to 70%. Bovine brucellosis is predominantly concentrated in the northern and southeastern regions, while infection in small ruminants is more widespread through the country [19]. Between 2014 and 2018, *Brucella* seroprevalence in Tunisia showed rates of 20.6% in aborted ruminants in the south, 55.6% in sheep and 21.8% in cattle in the center of the country, and 16.1% in aborted sheep across most regions [14,15,18]. Moreover, *Brucella* species were detected in 75% of dairy products, highlighting their relevance for public health [2].

Recent advances in next-generation sequencing (NGS) technologies have significantly improved the characterization of bacterial genomes. Short-read sequencing platforms, such as Illumina, provide highly accurate data with error rates below 1%, but their limited read length (<1 kb) often results in fragmented assemblies. Conversely, other NGS platforms like Oxford Nanopore Technologies (ONT) generate much longer reads (>10 kb), enabling better resolution of complex and repetitive regions. However, these long reads are prone to higher basecalling error rates (10–14%), which complicates downstream analyses. To merge the best of both platforms, hybrid assembly strategies have been developed to combine long reads with short reads for improved contiguity and for accurate error correction. Hybrid approaches enhance genome completeness, reduce assembly fragmentation, increase N50 values, and improve gene annotation [20,21,22,23,24,25]. In this study, we report the first hybrid assembly of *B. melitensis* animal isolates from Tunisia to generate a high-quality, near-complete genome.

Given the relevance of *B. melitensis* for animal and human health, and the lack of genomic data from Tunisian animal strains, this study was conducted to address these gaps by studying for the first time the genomes of *B. melitensis* from aborted sheep in Tunisia.

The aims of this work are as follows: (1) to assess the sensitivity of Tunisian *Brucella melitensis* isolates to a wide spectrum of antibiotics commonly used for treating brucellosis, evaluating their adequacy for human treatment; (2) to investigate antimicrobial resistance (AMR) and virulence genes in their genomes; and (3) to evaluate their genetic relationships with other Tunisian human strains, strains worldwide, and the vaccine strain Rev1. We hypothesized that the Tunisian animal strains are potentially closely related to the biovar 3 genotype and to other Tunisian human strains.

## 2. Materials and Methods

### 2.1. Origin of Brucella Strains

Data from passive surveillance were analyzed to isolate *Brucella* from ruminants. The cases included in this study were those of ruminants whose owners had voluntarily reported abortion during the lambing season. Two *B. melitensis* isolates were recovered from aborted sheep in two distinct regions of Tunisia: strain TATA in the southern region (Tataouine) and strain SBZ in the central region (Sidi Bouzid). In 2020, SBZ was isolated from a vaginal swab of a four-year-old ewe in late gestation, which had no history of abortion and had previously had two successful parturitions. In 2022, TATA was isolated from a vaginal swab of a six-year-old ewe which aborted during early gestation with no prior history of abortion nor parturition.

### 2.2. Isolation and Identification of Brucella Strains

All samples were processed under Biosafety Level 2+ and Level 3 conditions. The vaginal swabs were resuspended in 1 mL of sterile PBS, and 50 µL was spread on *Brucella*-selective agar with antibiotic supplement and on Columbia blood agar (Biolife italiana S.r.L., Milano, Italy) plates containing 5% sheep blood. In parallel, 100 µL of the suspension was inoculated into Trypticase Soy Broth (BIOKAR diagnostics, Beauvais, France) supplemented with *Brucella*-selective supplement (Oxoid Ltd., Basingstoke, UK). The cultures were incubated at 37 °C with a 5% CO_2_ atmosphere, and the plates were examined for presumptive colony-forming units (CFUs) of *Brucella* species on day 3 and daily for 2 weeks. Suspected CFUs were sub-cultured on blood agar for 48 h for subsequent molecular identification. Suspected CFUs of *Brucella* spp. were harvested in 450 μL nuclease-free water (Fluka, Lincolnshire, IL, USA) and inactivated at 100 °C for 10 min, to produce thermo-lysates. The suspensions were centrifuged at 13,000 rpm for 5 min and the DNA from the supernatant was collected for later molecular analyses. The detection of the genus *Brucella* and the species *B. melitensis* and *B. abortus* was performed by RT-PCR assays as described previously [2] using primer sets and probes according to Probert et al. [26]. The PCRs were considered positive at cycle threshold (Ct) values ≤ 37. Once confirmed, CFUs of *Brucella melitensis* were purified and stored at −80 °C in glycerol (15%).

### 2.3. Antibiotic Susceptibility Testing (AST)

Antibiotic susceptibility testing (AST) was conducted to assess the susceptibility of the isolates against nine antimicrobial agents: gentamicin, streptomycin, doxycycline, rifampicin, trimethoprim–sulfamethoxazole, ciprofloxacin, levofloxacin, ceftriaxone, and tetracycline. The selection of these antibiotics was based on the World Health Organization (WHO) guidelines for the treatment of human brucellosis [10]. The minimum inhibitory concentration (MIC) values were determined using the broth microdilution method, according to CLSI (version M45-A2) standards as described by Tscherne et al. [27]. The strain *Escherichia coli* ATCC 25922 was used as a quality control. AST work involving live *B. melitensis* was performed in a Biosafety Level 3 laboratory within a class II safety cabinet. After cultivation on blood agar (Biolife italiana S.r.L., Milano, Italy), the strains were suspended in 0.9% NaCl and adjusted to 0.5 McFarland standard units. For the final bacterial inoculum, 100 μL of the McFarland suspension was transferred to 11 mL of *Brucella* broth (BB, Becton Dickinson, East Rutherford, NJ, USA) as liquid culture medium (autoclaved for 15 min at 121 °C, subsequent pH adjustment to 7.0 ± 0.2). A bacterial inoculum of 100 μL was added to each well of a microdilution plate with the following setup of antimicrobials and concentrations: doxycycline from 0.015 to 8 mg L^−1^, gentamicin from 0.004 to 8 mg L^−1^, rifampicin from 0.012 to 4 mg L^−1^, streptomycin from 0.008 to 16 mg L^−1^, trimethoprim–sulfamethoxazole from 0.002/0.04 to 4/76 mg L^−1^, ceftriaxone from 0.015 to 2 mg L^−1^, ciprofloxacin from 0.002 to 4 mg L^−1^, levofloxacin from 0.004 to 4 mg L^−1^, and tetracycline from 0.25 to 8 mg L^−1^. Minimum inhibitory concentration (MIC) end points were read using an inverted mirror (VIZION, Thermo Fisher Scientific, Waltham, MA, USA) after incubation for 48 h at 36 ± 1 °C with 5% of CO_2_. For quality control, the number of CFUs in the bacterial inoculum was determined with a target of 5 × 10^5^ CFU mL^−1^ in the final culture broth. Therefore, the inoculum was diluted 1:1000 in 0.9% NaCl and streaked on Columbia agar plates. A range of 20 to 200 CFUs per plate was considered acceptable. The interpretation of MIC values was carried out according to the Clinical and Laboratory Standards Institute (CLSI) guidelines for *Brucella* species (Version M45-A2) [28].

### 2.4. DNA Extraction, Library Preparation, and Sequencing

The genomic DNA of the two *B. melitensis* isolates was extracted using the QIAamp DNA Mini Kit (QIAGEN, Hilden, Germany) according to the manufacturer’s instructions. The extracted DNA was quantified with the Qubit fluorometer (Qubit^TM^ DNA HS assay; Life Technologies, Thermo Fisher Scientific), and libraries for Oxford Nanopore Technologies (ONT, Oxford, UK) sequencing were prepared using the native barcoding kit (SQK-NBD114.24, ONT). Subsequently, MinION flow cells (R10.1.4 FLO-MIN106, ONT) and MinKNOW software v3.6.5 (ONT) were utilized for sequencing, data collection, basecalling, and barcoding. Additionally, the same DNA samples were processed for Illumina sequencing on a NextSeq 2000 (Illumina, San Diego, CA, USA) using P3 chemistry and 300 cycles (2 × 150 bp).

### 2.5. Whole-Genome Sequencing and Bioinformatic Analyses

Whole-genome sequencing (WGS) was performed using a hybrid approach combining Illumina and ONT reads. For quality control (QC), Illumina reads were assessed with Fast QC (v0.12.1) [29] and Multi QC tools (v1.16) [30]. Trimmomatic (v0.39) was employed for removing low-quality bases and adapters [31]. The quality of long reads obtained from ONT technologies was checked with NanoPlot (v1.41.6) [32]. Subsequently, ONT reads shorter than 1000 bases were filtered with filtlong (v0.2.1) [33]. Short and long reads that successfully passed the QC and filtering steps were integrated into a hybrid de novo assembler: Unicycler (v0.5.0) [34]. The quality and overall assembly statistics of the genomes were evaluated using QUAST (v5.2.0) [35]. Additionally, the hybrid assembly was visualized using Bandage (v0.8.1) [36]. Kraken (v2.1.3) [37] and Krona tools (v2.7.1) [38] were used for the taxonomy of reads and the detection of potential contamination. Genome completeness and the contamination of the assemblies were evaluated with CheckM (v1.1.6) [39]. The genome annotation was performed using a different approach involving Prokka (v1.14.6) [40] and Rapid Annotation using Subsystem Technology (RAST, https://rast.nmpdr.org/) (accessed on 1 January 2025) [41]. The visualization of two *B. melitensis* genomes was conducted using Proksee (https://proksee.ca/) (accessed on 28 June 2025) [42]. The genomes of *B. melitensis* were characterized using RAST and Anvio (v8) [41,43]. Multilocus sequence typing (MLST) profiles of the *Brucella* isolates were determined using the PubMLST database (https://pubmlst.org/) (accessed on 9 January 2025) [44]. Two typing schemes were applied: a 9-locus scheme targeting *gap*, *aroA*, glk, *dnaK*, *gyrB*, *trpE*, *cobQ*, *int_hyp*, and *omp25*; and a 21-locus scheme, which included the previous loci in addition to *prpE*, *caiA*, *csdB*, *soxA*, *leuA*, *mviM*, *fumC*, *fbaA*, *ddlA*, *putA*, *mutL*, and *acnA*.

The Tunisian genomes were compared with those of the reference strains *B. melitensis* bv. 1 16M (GCA_000007125.1) and *B. melitensis* bv. 3 Ether (GCA_000740355.1) and the vaccine strain *B. melitensis* Rev.1 (GCA_038420175.1) using anvio (v8) [43]. The sequence of amino acids from the gene clusters exclusive to the Tunisian strains was explored with blastp (NCBI) (https://www.ncbi.nlm.nih.gov/) (accessed on 12 January 2025). The detection of potential antimicrobial resistance (AMR) genes was performed using the Comprehensive Antibiotic Resistance Database (CARD) based on the Resistance Gene Identifier (RGI) (https://card.mcmaster.ca/) (accessed on 23 January 2025), AMRFinderPlus (v3.11.26) [45], and Abricate (v0.8.13) with the following databases: CARD [46], antimicrobial resistance database for mobile genetic elements (MEGARes) [47], and antimicrobial resistance gene database (ResFinder) [48]. Potential antimicrobial resistance mechanisms resulting from mutations were investigated by comparing the Tunisian strains (SBZ and TATA) with the reference strains *B. melitensis* 16M and *B. melitensis* bv. 3 Ether using the National Center for Biotechnology Information (NCBI) (https://www.ncbi.nlm.nih.gov/) (accessed on 23 January 2025). Potential virulence genes were identified using Abricate with the VFDB database [49] and Bakta (https://bakta.computational.bio/) (accessed on 23 May 2025) [50] to search for 7 additional *Brucella* putative virulence genes not listed in VFDB [51]. The genome sequences were submitted to the National Center for Biotechnology Information (NCBI) under BioProject ID PRJNA1247157 with the accession numbers SAMN47805342 for TATA and SAMN47805343 for SBZ.

A previously published core-genome-based multilocus sequence typing (cgMLST) assay [52] using SeqSphere+ software, v5.0.90 (Ridom GmbH, Münster, Germany), was conducted to determine the genetic relationships of Tunisian animal strains (TATA and SBZ) with other worldwide strains. For gene-by-gene comparison, the assay uses an identity of 90% for the target scan with a required 100% alignment to the respective reference genes. For cgMLST analysis, de novo hybrid assemblies were imported into SeqSphere+ as fasta files. The analysis included *B. melitensis* isolated from humans in Tunisia and different Mediterranean countries (Algeria, Morocco, Egypt, Italy, and France), a set of strains from an unusual brucellosis outbreak in Austria, and the biovar 3 reference strain Ether. As an out-group, the reference strain *B. melitensis* bv. 1 16M and the closely related vaccine strain (*B. melitensis* bv. 1 Rev1) were included. The phylogenetic relationships of all strains were visualized as a minimum spanning tree (MST) implemented in SeqSphere+ software.

## 3. Results

### 3.1. Brucella Growth, Identification, and Characterization

One isolate was recovered from each sample. After purification, typical CFUs on blood agar were observed after 48 h of incubation. CFUs showed a very small, glistening, smooth, round, and pin-point morphology. The DNA extracted from the two Tunisian isolates was positive in RT-PCR for *Brucella* species and *B. melitensis*, with Ct values ranging from 12 to 15. Whole-genome sequencing confirmed the presence of *Brucella* at the genus level and *B. melitensis* at the species level without any other species contamination. The completeness and contamination of the two *B. melitensis* assembled genomes were 99.33% and 0.95%, respectively. The genomes of the SBZ and TATA strains consisted of approximately 3.3 million base pairs, organized into two circular chromosomes, each with a G+C content of 57.2%. The SBZ and TATA strains contained 3350 and 3340 coding sequences, respectively (Table 1). Both Tunisian strains were classified as ST11 and ST89 based on MLST-9 and MLST-21 analyses, respectively.

**Figure 1:**
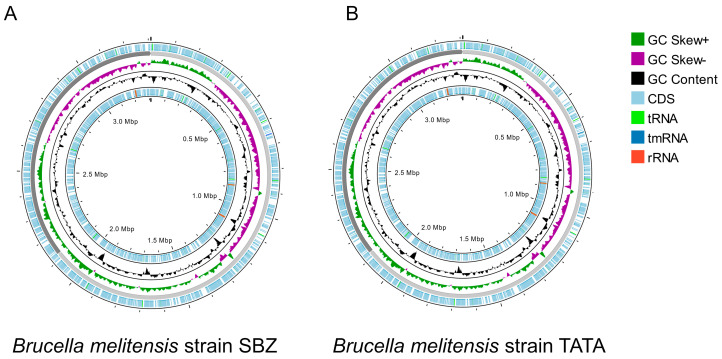
Overview of *Brucella melitensis* Tunisian strain genomes using Proksee [42]. (**A**) *Brucella melitensis* strain SBZ; (**B**) *Brucella melitensis* strain TATA. The map displays genomic features arranged in concentric rings. From the outermost to innermost rings: predicted coding sequences (CDS) on the forward (sense) and reverse (antisense) strands (light blue), transfer RNA (tRNA, light green), ribosomal RNA (rRNA, red), transfer–messenger RNA (tmRNA, dark blue), GC content plot (black), and GC Skew, with positive values shown in dark green (GC Skew+) and negative values in purple (GC Skew−). The scale markers indicate the genome size in megabase pairs (Mbp).

The general characteristics of these Tunisian strains of *B. melitensis* were also compared with those of the reference strains *B. melitensis* bv. 1 16M and *B. melitensis* bv. 3 Ether and the vaccine strain *B. melitensis* bv. 1 Rev.1 (Table 1). A genomic overview of the TATA and SBZ strains is presented in Figure 1.

**Figure 2:**
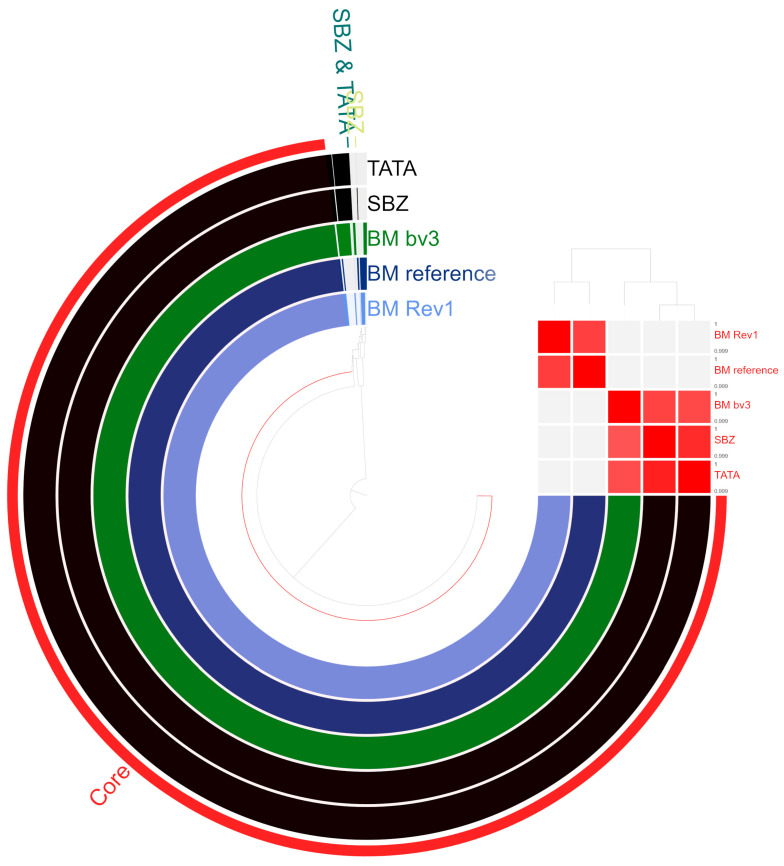
Genomic comparison between the Tunisian strains (SBZ and TATA in black) and the reference strains *B. melitensis* bv. 1 16M (BM ref in dark blue), *B. melitensis* bv. 1 Rev.1 (BM Rev1 in light blue), and bv. 3 Ether (BM bv3 in green). Heat map showing differences at average nucleotide identity (ANI) values over 99.9%.

The genomes of the two Tunisian strains are classified into 27 categories within 407 subsystems. The five most prominent functional categories in both strains are amino acids and derivatives; carbohydrates; protein metabolism; cofactors, vitamins, prosthetic groups, and pigments; and respiration. The subsystem categories of the two Tunisian *B. melitensis* strains are compared with those of the reference strains *B. melitensis* bv. 1 16M and *B. melitensis* bv. 3 Ether, and the vaccine strain *B. melitensis* bv. 1 Rev.1 in Table S1. The average nucleotide identity of over 99% between the genomes of the Tunisian strains (TATA and SBZ) and the reference strains is evidenced in Figure 2. Five gene clusters were exclusive for SBZ and TATA and represented with 100% identity by four hypothetical proteins: hypothetical protein BMEI1701, hypothetical protein B984_01900, and two gene clusters that were unique for the strain SBZ, namely, a rhodocoxin reductase and the hypothetical protein DK60_167.

### 3.2. Antibiotic Susceptibility Testing (AST)

Antibiotic susceptibility testing (AST) was interpreted according to the recommended reference ranges for *Brucella* spp. for all antibiotics. The exception was rifampicin, which was based on the reference range for *Haemophilus influenzae* as defined by CLSI. Considering the MIC values, both *B. melitensis* isolates were susceptible to gentamicin, doxycycline, rifampicin, streptomycin, and trimethoprim–sulfamethoxazole (Table 2).

### 3.3. Identification of AMR and Virulence-Associated Genes

The in silico analysis of AMR genes of the two genomes of Tunisian *B. melitensis* isolates identified the multiple peptide resistance factor (*mprF*) gene, alongside three additional genes: *qacG*, *fosXcc*, and *adeF*. The presence of *mprF* was also identified using Abricate. Additionally, the analysis with Abricate and AMRfinderPlus software revealed the presence of RND-family efflux genes, *bepC*, *bepD*, *bepE*, *bepF*, and *bepG*, all exhibiting more than 99% identity (Table 3). No classical AMR genes were identified by these tools. However, annotating *B. melitensis* genomes revealed the presence of several genes responsible for crucial genomic functions and, in some cases, for also conferring antimicrobial resistance, such as sulfamethoxazole (*folP*), rifampicin (*rpoB*), streptomycin (*rpsL*, *rsmG*), and fluoroquinolones (*gyrA*, *gyrB*, *parC*). Compared to the reference strains *B. melitensis* bv. 1 16M and *B. melitensis* bv. 3 Ether, a single point mutation was identified in the *parC* gene of both *B. melitensis* isolates at position Ala100, where alanine was substituted by threonine.

A total of 74 potential virulence genes (Table 4, Table S2 and Figure S1) were identified in the two *B. melitensis* isolates (TATA and SBZ) and the reference strains (*B. melitensis* bv. 1 16M and *B. melitensis* bv. 3), including genes associated with lipopolysaccharide synthesis (n = 34), the type IV secretion system and its effectors (n = 26), adhesines (n = 4), Tir domain-containing protein (n = 2), the two-component system BvrR/BvrS (n = 2), cyclic β-1,2-glucan production (n = 1), peptidoglycan (n = 1), the biosynthesis of glycine betaine (n = 1), outer membrane protein (n = 1), urease (n = 1), and proline racemases (n = 1). Through the use of Abricate, most genes showed over 99% similarity between the isolates, except for *bspL* and *bigA*, which exhibited slightly lower similarity percentages.

### 3.4. Core-Genome-Based MLST (cgMLST)

**Figure 3:**
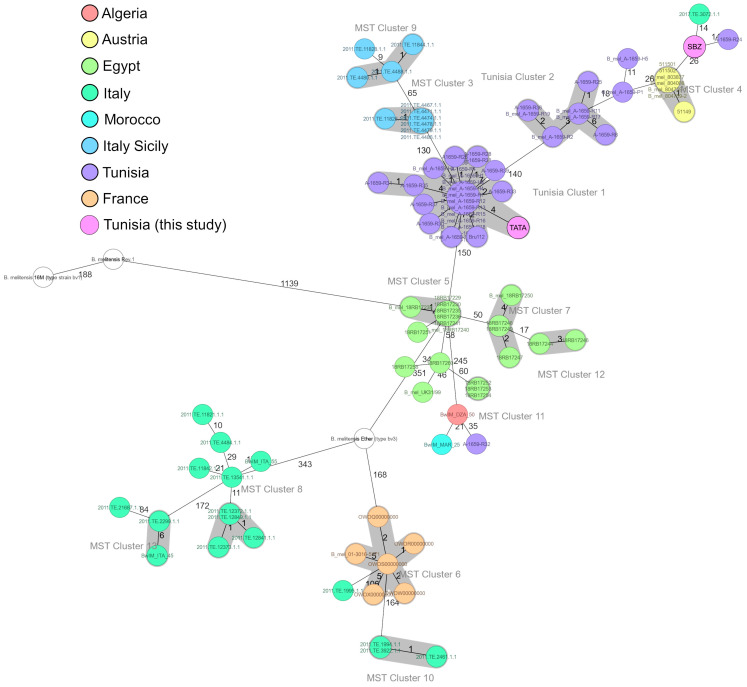
Minimum spanning tree (MST) from core-genome multilocus sequence typing (cgMLST) of *Brucella melitensis* strains.

In the minimum spanning tree (MST) analysis (Figure 3), the TATA and SBZ strains were grouped with the other *B. melitensis* biovar 3 strains. The strains of biovar 1 (16M, Rev1) displayed a more considerable genetic distance exceeding 1000 allelic differences.

The MST further highlights the genetic divergence between the animal strains SBZ and TATA, which were located in two distinct groups. The TATA strain clusters within Tunisian Cluster 1, which consists of closely related human isolates exhibiting no more than four allelic differences. This cluster shows the closest genetic relationship to Tunisian Cluster 2, with a distance of 140 discriminating alleles. Tunisian Cluster 1 is separated by 150 alleles from Egyptian isolates and by 130 alleles from Italian (Sicilian) isolates.

In contrast, the SBZ strain was situated near Tunisian group 2, with an allelic difference of 26 from the Austrian samples. The SBZ strain is most closely related to one Italian and one Tunisian isolate, each differing by 14 alleles, and is separated by 26 alleles from the Austrian isolates.

## 4. Discussion

This study reports the first isolation of *B. melitensis* from two aborted sheep in distinct regions of Tunisia (Tataouine and Sidi Bouzid) using microbiological culture, which remains the gold standard for confirmatory diagnosis [53]. It also provides the first genomic characterization of these animal isolates through a hybrid whole-genome sequencing approach.

Real-Time PCR and WGS were employed for *Brucella* identification, showing consistent results at both the genus and species levels. WGS, combined with multiplex PCR, proved to be a reliable and accurate approach for strain classification, with the potential to replace classical biotyping and to reduce the risk of laboratory-acquired infection [54,55].

WGS analysis revealed a highly conserved genome structure in the Tunisian strains comparable to *B. melitensis* Ether bv. 3 and *B. melitensis* 16M. Several subsystems in the genome structure are involved in fundamental cellular processes essential for bacterial survival [56]. An average nucleotide similarity over 99% between the genomes of the Tunisian strains and the *B. melitensis* references was evidenced. Despite this high conservation, five gene clusters were unique to the SBZ and TATA strains, representing hypothetical proteins. These hypothetical proteins may provide critical insights into the biology and pathogenesis of *Brucella* [57]. These findings may lay the groundwork for future comparative genomic and functional studies.

MLST-9 typing revealed that the two Tunisian isolates belonged to sequence type 11 (ST11). This sequence type has been previously documented in Mediterranean strains from Egypt, Morocco, Algeria, and Italy [58].

Our results provide the first confirmed evidence of susceptibility to five antibiotics in *Brucella* species from Tunisia. These findings suggest that gentamicin, doxycycline, rifampicin, streptomycin, and trimethoprim–sulfamethoxazole remain effective against *B. melitensis* in Tunisia, aligning with previously reported susceptibility patterns in Iran [54], Egypt [55], Norway [59], and Turkey [60]. In Tunisia, brucellosis treatment follows the World Health Organization (WHO) guidelines. Unlike in humans, antibiotic treatment in livestock is not recommended [10]. However, antibiotics are frequently used in veterinary medicine to treat and prevent diseases, as well as in livestock production for food consumption in developing countries, to enhance animal welfare and promote growth [61]. The misuse of antibiotics, often in the absence of veterinary supervision, further exacerbates the issue, as treatments may be administered improperly or without appropriate diagnosis.

The knowledge gap in Tunisia is primarily due to the significant biosafety challenges associated with handling *Brucella* species, which are highly infectious, are readily aerosolized, and pose a considerable risk of laboratory-acquired infections [59]. The manipulation of these pathogens requires Biosafety Level 3 (BSL-3) facilities, which remain limited in Tunisia. The antibiotic susceptibility of our animal strains may be explained by the implementation of control measures, such as the culling of infected animals in Tunisia. Indeed, infected livestock serve as the primary reservoir of *Brucella*, and the removal of infected individuals from herds reduces the likelihood of prolonged bacterial exposure to antibiotics, thereby limiting the selective pressure that could drive the development of AMR. Additionally, the intracellular lifestyle of *Brucella* hinders the penetration of various antimicrobials and may impair resistance development [55]. However, higher AMR in the phenotype of *Brucella* has become evident in recent years. Resistance to gentamicin, doxycycline, rifampicin, streptomycin, and trimethoprim–sulfamethoxazole has been detected in *Brucella* isolates from Iran, Egypt, Turkey, India, and south Europe [54,55,62,63,64,65]. Therefore, ongoing surveillance is essential to monitor the potential development of resistance and ensure the efficacy of these antibiotics for human and animal treatment.

Ciprofloxacin, levofloxacin, ceftriaxone, and tetracycline were tested using the broth microdilution method in accordance with CLSI guidelines. However, specific clinical breakpoints for these agents against *Brucella* spp. are currently not available in CLSI standards. Tscherne et al. [27] compared different parameters for broth microdilution methods, including those outlined in the guidelines of CLSI and the European Committee on Antimicrobial Susceptibility Testing. Specifically, the study evaluated the two media employed in these protocols: *Brucella* broth (BB) by CLSI, and Cation-Adjusted Mueller–Hinton Broth (CAMHB) by EUCAST. Their findings showed that for ciprofloxacin and levofloxacin, the choice of medium had no significant effect on MIC values for the strains tested. These results enable us to make an assumption regarding the interpretation of our own results. When compared with the recently updated EUCAST MIC breakpoints for *B. melitensis* [66] (susceptible ≤0.001 µg mL^−1^; resistant >1 µg mL^−1^), the results for our isolates (MIC of 0.5 µg mL^−1^) could indicate an intermediate resistance to both fluoroquinolones. This intermediate resistance may evolve into full resistance over time, especially considering that resistance to fluoroquinolones has been reported in isolates from India [64], Egypt [62,67], and Mexico [68].

Ceftriaxone and tetracycline were not assessed in the study by Tscherne et al. [27]. However, applying the same extrapolation to the measured MIC value for ceftriaxone of 0.5 µg mL^−1^ for both the TATA and SBZ strains points toward potential susceptibility (≤2 µg mL^−1^). Similarly, the MIC value for tetracycline of 0.25 µg mL^−1^ for both strains may indicate susceptibility considering the EUCAST threshold of 0.5 µg mL^−1^. These findings suggest that both ceftriaxone and tetracycline could be effective therapeutic options against the isolates tested, provided that testing according to EUCAST and CLSI are comparable for these strains. In support of this assumption, similar susceptibility patterns have already been reported in Iran [54], Egypt [55,67], and southern Europe [46].

WGS analysis revealed the absence of classical antimicrobial resistance genes, suggesting that our strains are susceptible to most antibiotics as also evidenced phenotypically. One mutation was identified in the genomes of *B. melitensis* isolates when compared to the reference strains *B. melitensis* 16M and *B. melitensis* bv. 3 Ether. This mutation was located in the *parC* gene, which is associated with fluoroquinolone resistance [69,70]. Notably, it differs from previously reported mutations known to confer resistance in *B. melitensis* [59]. Further functional analyses are necessary to assess this mutation’s potential role in antimicrobial resistance.

The identification of multiple peptide resistance factors is noteworthy. For example, *mprF* plays an essential role in resistance to cationic antibiotics such as gentamicin, moenomycin, vancomycin, methicillin, oxacillin, bacitracin, and beta-lactams. However, none of our isolates exhibited resistance to gentamicin. The specific role of *mprF* in virulence and antimicrobial resistance in *Brucella* remains unclear [54,55,71]. The genes for *bep* were reported to increase resistance to certain antibiotic compounds, such as tetracycline, doxycycline, chloramphenicol, and ciprofloxacin, in *B. suis*. Hence, none of the *B. melitensis* isolates showed resistance to doxycycline. The presence of *mprF* and *bepCDEFG* genes has been systematically reported in classical pathogenic *Brucella* species, including *B. melitensis*, *B. abortus*, and *B. suis*, across various studies conducted in Tunisia, Iran, Egypt, Turkey, and India [54,55,63,64,72]. Moreover, recent genomic studies have confirmed that these genes are also consistently present in non-classical *Brucella* species, such as marine species *B. ceti* and *B. pinnipedialis* [73,74]. Despite their widespread presence in both classical and non-classical *Brucella* species, no studies to date have demonstrated a direct functional association between these genes and antimicrobial resistance in *Brucella* species [54,55,64]. This aligns with our findings, underscoring the need for caution in the interpretation of the presence of these genes, which may reflect conserved genomic traits rather than actual resistance mechanisms. Other efflux pump genes including *adeF* (RND efflux pmp), *qacG* (SMR efflux pump), and *fosXCC* were also reported in *B. melitensis* from north-eastern Ethiopia [75]. However, the presence of these genes does not necessarily indicate phenotypic resistance. Indeed, previous research has indicated that these potential AMR genes are broadly distributed among *Brucella* species across various geographic regions without a consistent association with phenotypic AMR [54,64,75].

The in silico analysis of virulence genes in the two Tunisian *B. melitensis* isolates showed a common pattern with the reference strains of biovars 1 and 3. Unlike many pathogens, *Brucella*, classified as an intracellular bacterium, lacks classical virulence factors such as proteases, cytolysins, exotoxins, capsules, exoenzymes, virulence plasmids, and fimbriae or pili [54,76]. However, the pathogenesis of *Brucella* spp. resides in their ability to invade, survive, and replicate in diverse phagocytic and non-phagocytic cell types, leading to chronic infections [76,77].

In this study, the same genes with the potential for virulence were identified in our isolates and reference strains. This finding suggests a conserved virulence gene repertoire and highlights the genomic stability of *Brucella* species. The high frequency of these genes highlights the pathogenic potential of the *Brucella* strains specifically in the Tataouine and Sidi Bouzid regions of Tunisia and aligns with the clinical context, as all isolates were recovered from abortion cases. The potential virulence factors identified in this study are similar to those of a recent study conducted in Tunisia on B. melitensis human isolates [72]. Additionally, most of these have also been previously documented in isolates from Iran [54], Egypt [55,78], and Italy [79].

Two classes of adhesines have been identified in both strains: the BigA and BigB proteins (encoded by *bigA* and *bigB* genes) that contain an Ig-like domain, binding to cell adhesion molecules in epithelial cells, and the monomeric autotransporters BmaA and BmaB/OmA (encoded by *bmaA* and *bmaB*/*omA* genes), binding to ECM components, epithelial cells, osteoblasts, synoviocytes, and trophoblasts [77]. In addition to adhesines, the complete LPS acts as a key virulence factor by facilitating adherence and invasion through the connection of the O-chain with lipid rafts on the macrophage surface [76]. Moreover, it plays a pivotal role in evading the host’s innate immune defense, demonstrated by weak myeloid differentiation-2 (MD2) binding and low endotoxicity. Additionally, it limits complement deposition, activation, and neutrophil-mediated killing [80]. On study revealed that a majority of virulence-associated genes in *B. melitensis* isolates are implicated in the processes of LPS synthesis, maturation, and functionality. In addition, several other potential virulence factors with crucial roles in immune evasion and intracellular survival were identified [81]. The TIR domain-containing proteins BtpA and BtpB interfere with Toll-like receptor (TLR) signaling to temper the host inflammatory response [67]. Moreover, the type IV secretion system (T4SS), governed by the *virB* gene and its effectors, plays a vital role in bacterial adherence to host cells, cell entry, intracellular trafficking, and survival [82]. The BvrR/BvrS two-component system regulates the expression of *virB* by the positive stimulation of *vjbR* transcription [83]. Finally, the *cβg* gene, encoding cyclic ß-1,2-glucans (CßGs), interferes with cellular trafficking by acting on lipid rafts on host cell membranes, preventing the phagosome–lysosome fusion cycle [84]. Among other important virulence factors, urease (e.g., *ure1* and *ure2*) plays a crucial role in the resistance of *Brucella* species in low-pH conditions [81,85]. The *Brucella* proline racemase protein A (PrpA) functions as a B lymphocyte mitogen, promoting the secretion of interleukin-10 (IL-10), which downregulates Th1 immune responses and contributes to the immunosuppressive environment characteristic of chronic brucellosis [85]. Betaine aldehyde dehydrogenase (BADH), encoded by the *betB* gene, converts betaine aldehyde into glycine betaine using NAD(P)+. Glycine betaine acts as an osmoregulatory molecule, enhancing *Brucella* survival under osmotic stress and aiding in the synthesis of phosphatidylcholine (PC), a critical component of the outer membrane involved in intracellular trafficking and persistence [85,86].

Our animal strains were clustered together with other *B. melitensis* bv. 3 strains and showed a more distant relationship with biovar 1 strains (16M, Rev.1). This finding suggests that the strains are potentially closely related to previously characterized bv. 3 strains, indicating the possible circulation of a similar lineage in the Tunisian sheep population. However, this finding is solely based on phylogenetic characterization and needs to be verified by phenotypical comparison. The TATA strain is part of Tunisian Cluster 1, while the SBZ strain is located near the second-largest Tunisian group but exhibits the highest genetic similarity to a single human isolate from Tunisia and Italy. Notably, the close genetic relation to isolates from a brucellosis outbreak in Austria suggests a potential epidemiological link.

The presence of the TATA strain within Tunisian Cluster 1 may implicate TATA as a potential source of human infection. Interestingly, the human strains of *B. melitensis* in this cluster were isolated in the same year (2017) and showed only four allelic differences. The absence of data from an animal strain detected before 2017 further emphasizes the potential of the TATA strain to circulate in livestock undetected before 2022, contributing to zoonotic transmissions to humans. Additionally, the isolated animal strain SBZ is very close (14 allelic differences) to the Tunisian human strain 1659-R24 isolated in 2016, suggesting that they are closely related. These findings reinforce the role of livestock as a reservoir for human infections. The absence of major mutations in *Brucella* animal strains over several years suggests significant genetic stability, indicating optimal adaptation to their host. This stability may contribute to the persistence of these strains in the environment and facilitate their continuous transmission within animal populations. Such adaptation could also play a key role in the epidemiological dynamics of brucellosis, highlighting the need for enhanced surveillance to better understand and control the spread of the disease. The animal strains of this study were isolated from the south and the center of Tunisia, whereas the Tunisian human strains were found to circulate mainly in the north. Notably, historical outbreaks further support the hypothesis of interregional transmission. Significant human and animal brucellosis epidemics have been reported in both the southern and northern regions of Tunisia [19,72], suggesting that these areas may serve as important reservoirs for *Brucella* circulation. The observed genetic relatedness between the Tunisian animal and human strains may indicate the movement of infected animals or contaminated animal-derived products between the south and the north, particularly during religious events (Eid al-Adha, Ramadan, Hajj) and social gatherings (e.g., weddings). Furthermore, human brucellosis has been strongly associated with the consumption of raw milk and its derivatives in Tunisia: 93.3% of clinical human brucellosis (CHB) cases were linked to the ingestion of unpasteurized dairy products [18], and 77% of neurobrucellosis cases were attributed to foodborne transmission [87]. More recently, a study in northern Tunisia revealed a high contamination rate of 75% for *Brucella* spp. DNA in unpasteurized dairy products [2].

Phylogenetic analysis further provides valuable insights into the evolutionary relationships between Tunisian and international *Brucella* strains. Tunisian Cluster 1 included the animal strain TATA, which is closely related to Tunisian Cluster 2 and strains from Italy and Egypt, suggesting historical and contemporary trade routes as potential pathways for *Brucella* dissemination. The long-standing commercial exchanges between Italy and North Africa, dating back to the Roman Empire and persisting through the colonial period, may have facilitated the spread of *Brucella* species across the Mediterranean basin [72].

The Tunisian strains and Austrian strains likely share a common ancestor but have evolved independently over time, possibly due to geographical separation and distinct epidemiological dynamics. Furthermore, the animal strain SBZ is genetically closer to an Italian strain. This may be explained by the importation of Italian and Austrian isolates from Tunisia. This connection was previously reported in an Austrian study, which demonstrated that *B. melitensis* isolates collected between 2005 and 2015 were closely related to a phylogenetic branch that included strains from Egypt and Italy [72].

## 5. Conclusions

Whole-genome sequencing (WGS) provides valuable insights into the genetic potential for antimicrobial resistance (AMR) and virulence in *Brucella melitensis*. No differences were observed in the distribution of potential AMR and virulence genes among the analyzed Tunisian strains and between these strains and the reference genomes of *B. melitensis* bv. 1 16M and *B. melitensis* bv. 3 Ether. The tested *Brucella* isolates remained susceptible to five antibiotics used for human treatment. Further research at the proteomic and transcriptomic levels is essential to elucidate the mechanisms underlying resistance and virulence in *B. melitensis* and to better understand and mitigate this zoonotic disease. WGS has significantly contributed to the understanding of the epidemiology of *B. melitensis* animal strains in Tunisia, shedding light on the phylogenetic relationships between circulating animal and human strains in Tunisia and neighboring countries. This represents a crucial step in delineating the evolutionary trajectory of *B. melitensis* in the region. Nonetheless, additional genomic data and strains from diverse geographical regions are required for a better understanding and more-robust interpretations of the phylogenetic relationships identified. Finally, strengthening vaccination programs and enhancing public awareness about the health and economic impacts of brucellosis, particularly in endemic areas, are critical for the success of prevention and control strategies.

## Figures and Tables

**Table 1:** General characteristics of the Tunisian strains (SBZ and TATA), *Brucella melitensis* bv. 3 Ether, *B. melitensis* bv. 1 16M, and *B. melitensis* bv. 1 Rev.1 according to Anvio and RAST tools [41,43].

Stats of Strains	SBZ	TATA	*B. melitensis* bv. 3 Ether	*B. melitensis* bv. 1 16 M	*B. melitensis* bv. 1 Rev.1
Genome size (bp)	3,310,738	3,310,809	3,310,727	3,294,931	3,299,187
Chromosome I size (bp)	2,122,905	2,122,935	2,122,766	2,117,144	2,121,370
Chromosome II size (bp)	1,187,833	1,187,874	1,187,961	1,177,787	1,177,817
Num genes (Prodigal)	3153	3152	3159	3152	3148
L50 value	1	1	1	1	1
L75 value	2	2	2	2	2
L90 value	2	2	2	2	2
N50 value (bp)	2,122,905	2,122,935	2,122,766	2,117,144	2,121,370
N75 value (bp)	1,187,833	1,187,874	1,187,961	1,177,787	1,177,817
N90 value (bp)	1,187,833	1,187,874	1,187,961	1,177,787	1,177,817
GC content (bp)	57.2	57.2	57.2	57.2	57.2
Number of subsystems	407	407	304	303	303
Number of coding sequences	3350	3340	3326	3326	3322

bp: base pairs; Num: numbers.

**Table 2:** Minimum inhibitory concentration (MIC) ranges, values, and breakpoints of Tunisian *B. melitensis* strains (TATA and SBZ) against nine antibiotics according to CLSI version M45-A2.

Antibiotic	MIC Range (µg/mL)	MIC Values of SBZ Strain (µg/mL)	MIC Values of TATA Strain (µg/mL)		MIC Interpretive Criteria (µg/mL)		Phenotype of SBZ Strain	Phenotype of TATA Strain
				S≤	I	R≥		
Gentamicin	0.004–8	0.5	0.5	4	-	-	S	S
Streptomycin	0.008–16	2	4	16 ^b^	-	-	S	S
Doxycycline	0.015–8	0.06	0.06	1	.	-	S	S
Rifampicin ^a^	0.012–4	1	1	1	2	4	S	S
Trimethoprim–Sulfamethoxazole	0.002/0.04 to 4/76	0.5	0.5	2/38	-	-	S	S
Ceftriaxone	0.015–2	0.5	0.5	ND *	ND *	ND *	-	-
Ciprofloxacin	0.002–4	0.5	0.5	ND *	ND *	ND *	-	-
Levofloxacin	0.004–4	0.5	0.5	ND *	ND *	ND *	-	-
Tetracycline	0.25–8	<0.25	<0.25	ND *	ND *	ND *	-	-

Standard breakpoints according to CLSI: S: sensitive; I: intermediate; and R: resistant. ^a^ CLSI interpretation of *Haemophilus influenzae* (fastidious bacteria). ^b^ For incubation conditions with 5% CO_2_. * Not described by CLSI standards.

**Table 3:** Summary of potential antimicrobial resistance (AMR) gene identification in *B. melitensis* isolates using ABRicate, AMRfinderPlus, and the CARD.

		Identification of AMR Genes (% Identity)					
		Abricate (MEGARes)		AMRfinderPlus (BLASTX/(PARTIALX)		CARD (RGI)	
Type	AMR Genes	SBZ Strain	TATA Strain	SBZ Strain	TATA Strain	SBZ Strain	TATA Strain
Resistance–Nodulation–Cell Division (RND) efflux pumps (Drug_and_biocide resistance)	*bepC*	99.64	99.64	99.78	99.78	-	-
	*bepD*	99.92	99.92	99.75	99.75	-	-
	*bepE*	99.84	99.84	99.62	99.62	-	-
	*bepG*	99.29	99.29	99.38	99.38	-	-
	*bepF*	98.79	98.87	98.54	98.54	-	-
RND efflux pump (Fluoroquinolone and tetracycline resistance)	*adeF*	-	-	-	-	46.73	46.73
Antibiotic target alteration with defensin resistance (Cationic antibiotic resistance)	*mprF*	99.58	99.58	-	-	99.54	99.54
Antibiotic inactivation with Fosfomycin thiol transferase (Phosphonic acid antibiotic resistance)	*fosxcc*	-	-	-	-	54.81	54.81
Small multidrug resistance (SMR) antibiotic efflux pump/disinfecting agent and antiseptic resistance	*qacG*	-	-	-	-	42.31	42.31

**Table 4:** Potential virulence factors and their associated genes identified in two Tunisian *Brucella melitensis* genomes (TATA and SBZ), as well as in *Brucella melitensis* bv. 1 16M and *B. melitensis* bv.3.

Virulence Factors	Related Genes
Adhesines	*bigA*, *bigB*, *bmaB*/*omA*, *bmaC*
Lipopolysaccharide (LPS)	*gmd*, *per*, *pgm*, *pmm*, *manAoAg*, *manCoAg*, *wzm*, *wzt*, *wbkB*, *wbkC*, *wbkA*, *wbpZ*, *wbpL*, *lpsA*, *acpXL*, *wboA*, *wbdA*, *lpsB*, *lpcC*, *manCcore*, *manBcore*, *fabZ*, *lpxA*, *lpxC*, *lpxD*, *lpxB*, *lpxE*, *lpxK*, *KdsA*, *kdsB*, *waaA*/*kdtA*, *htrB*, *manA*, *perA*
Type IV secretion system (*VirB*)	*virB1*, *virB2*, *virB3*, *virB4*, *virB5*, *virB6*, *virB7*, *virB8*, *virB9*, *virB10*, *virB11*, *virB12*
Type IV secretion system effectors	*vceA*, *vceC*, *ricA*, *BPE005*, *BPE043*, *BPE275*, *BPE123*, *sepA*, *bspA*, *bspB*, *BspC*, *bspE*, *bspF*, *bspL*
TIR domain-containing protein immune evasion	*BtpA*/*Btp1*/*TcpB* and *BtpB*
Two-component system BvrR/BvrS (TCS BvrRS)	*bvrR* and *bvrS*
CβG (cyclic β-1,2 glucan)	*Cgs*
Peptidoglycan	*mviN*
Biosynthesis of glycine betaine	*betB*
Outer membrane protein	*omp19*
Urease	*Ure*
Proline racemases	*prpA*

## Data Availability

The original contributions presented in this study are included in the article and Supplementary Materials. Further inquiries can be directed to the corresponding author.

## Supplementary Materials

The following supporting information can be downloaded at https://www.mdpi.com/article/10.3390/microorganisms13071651/s1, Table S1: Subsystem categories assigned to genes predicted in genome of Tunisian strains, *B. melitensis* bv. 3 Ether, and *B. melitensisis* strain Rev.1 based on RAST Server; Table S2: The results of in silico analysis of virulence-associated genes in two *B. melitensis* isolates from aborted sheep in Tunisia via Abricate; Figure S1: Virulence factors and their associated genes identified in Tunisian animal *Brucella melitensis* genomes.

## References

[1] Daugaliyeva A., Daugaliyeva S., Kydyr N., Peletto S. (2024). Molecular typing methods to Characterize *Brucella* spp. from animals: A Review. Vet. World.

[2] Béjaoui A., Ben Abdallah I., Maaroufi A. (2022). *Brucella* spp. Contamination in Artisanal Unpasteurized Dairy Products: An Emerging Foodborne Threat in Tunisia. Foods.

[3] Lokamar P.N., Kutwah M.A., Atieli H., Gumo S., Ouma C. (2020). Socio-economic impacts of brucellosis on livestock production and reproduction performance in Koibatek and Marigat regions, Baringo County, Kenya. BMC Vet. Res..

[4] Shekhar C. (2018). Impact of brucellosis on health and economy. Vet. Sci. Res. J..

[5] Ibarra M., Campos M., Ibarra C., Gladys U., Huera D., Gutiérrez M., Chamorro A., Núñez L. (2023). Financial Losses Associated with Bovine Brucellosis (*Brucella abortus*) in Carchi-Ecuador. Open J. Anim. Sci..

[6] González-Espinoza G., Arce-Gorvel V., Mémet S., Gorvel J.-P. (2021). *Brucella*: Reservoirs and Niches in Animals and Humans. Pathogens.

[7] Aragón-Aranda B., De Miguel M.J., Lázaro-Antón L., Salvador-Bescós M., Zúñiga-Ripa A., Moriyón I., Iriarte M., Muñoz P.M., Conde-Álvarez R. (2020). Development of attenuated live vaccine candidates against swine brucellosis in a non-Zoonotic *B. Suis* Biovar 2 background. Vet. Res..

[8] Sadaqat M.H. (2023). pathogenesis, clinical manifestations, treatment and prevention of brucellosis in humans and animals. Afghan. J. Basic. Med. Sci..

[9] Lindahl-Rajala E., Hoffman T., Fretin D., Godfroid J., Sattorov N., Boqvist S., Lundkvist Å., Magnusson U. (2017). Detection and characterization of *Brucella* spp. in bovine milk in small-scale urban and peri-urban farming in Tajikistan. PLoS Negl. Trop. Dis..

[10] Corbel M.J., Food and Agriculture Organization of the United Nations, World Health Organization (2006). Brucellosis in Humans and Animals.

[11] Rossetti C.A., Maurizio E., Rossi U.A. (2022). Comparative Review of Brucellosis in Small Domestic Ruminants. Front. Vet. Sci..

[12] Azam S., Rao S.B., Jakka P., NarasimhaRao V., Bhargavi B., Gupta V.K., Radhakrishnan G. (2016). Genetic Characterization and Comparative Genome Analysis of *Brucella melitensis* Isolates from India. Int. J. Genom..

[13] Hayoun M.A., Muco E., Shorman M. (2024). Brucellosis. StatPearls.

[14] Barkallah M., Gharbi Y., Zormati S., Karkouch N., Mallek Z., Gautier M., Gdoura R., Fendri I. (2017). A Mixed methods study of ruminant Brucellosis in central-eastern Tunisia. Trop. Anim. Health Prod..

[15] Guesmi K., Kalthoum S., Mamlouk A., Baccar M.N., BelHajMohamed B., Hajlaoui H., Toumi A., Cherni J., Seghaier C., Messadi L. (2023). Seroprevalence of zoonotic abortive diseases and their associated risk factors in Tunisian sheep. BMC Vet. Res..

[16] Selmi R., Mamlouk A., Belkahia H., Ben Yahia H., Abdelaali H., Jemli M.-H., Ben Said M., Messadi L. (2024). Serological and molecular survey of brucellosis and chlamydiosis in dromedary Camels from Tunisia. Comp. Immunol. Microbiol. Infect. Dis..

[17] Battikh H., Berriche A., Zayoud R., Ammari L., Abdelmalek R., Kilani B., Tiouiri Ben Aissa H., Zribi M. (2021). Clinical and laboratory features of brucellosis in a university hospital in Tunisia. Infect. Dis. Now..

[18] Khamassi Khbou M., Htira S., Harabech K., Benzarti M. (2018). First case-control study of zoonotic brucellosis in Gafsa district, Southwest Tunisia. One Health.

[19] Guesmi K., Kalthoum S., Belhaj Mohamed B., Ben Aicha I., Hajlaoui H., Hrabech K. (2020). Bilan de la brucellose animale et humaine en Tunisie: 2005–2018. Bull. Zoosanit..

[20] Eleiwa A., Nadal J., Vilaprinyo E., Marin-Sanguino A., Sorribas A., Basallo O., Lucido A., Richart C., Pena R.N., Ros-Freixedes R. (2024). Hybrid assembly and comparative genomics unveil insights into the evolution and biology of the red-legged partridge. Sci. Rep..

[21] Ruan Z., Wu J., Chen H., Draz M.S., Xu J., He F. (2020). Hybrid Genome Assembly and Annotation of a Pandrug-Resistant Klebsiella Pneumoniae Strain Using Nanopore and Illumina Sequencing. Infect. Drug Resist..

[22] Pfeiffer F., Gröber C., Blank M., Händler K., Beyer M., Schultze J.L., Mayer G. (2018). Systematic evaluation of error rates and causes in short samples in next-generation sequencing. Sci. Rep..

[23] Ma X., Shao Y., Tian L., Flasch D.A., Mulder H.L., Edmonson M.N., Liu Y., Chen X., Newman S., Nakitandwe J. (2019). Analysis of error profiles in deep next-generation sequencing data. Genome Biol..

[24] Magi A., Semeraro R., Mingrino A., Giusti B., D’Aurizio R. (2017). Nanopore sequencing data analysis: State of the art, applications and challenges. Brief. Bioinform..

[25] Weissensteiner M.H., Pang A.W.C., Bunikis I., Höijer I., Vinnere-Petterson O., Suh A., Wolf J.B.W. (2017). Combination of short-read, long-read, and optical mapping assemblies reveals large-scale tandem repeat arrays with population genetic implications. Genome Res..

[26] Probert W.S., Schrader K.N., Khuong N.Y., Bystrom S.L., Graves M.H. (2004). Real-Time Multiplex PCR Assay for Detection of *Brucella* spp., *B. abortus*, and *B. melitensis*. J. Clin. Microbiol..

[27] Tscherne A., Mantel E., Boskani T., Budniak S., Elschner M., Fasanella A., Feruglio S.L., Galante D., Giske C.G., Grunow R. (2022). Adaptation of *Brucella melitensis* Antimicrobial Susceptibility Testing to the ISO 20776 Standard and Validation of the Method. Microorganisms.

[28] Clinical and Laboratory Standards Institute (2016). Methods for Antimicrobial Dilution and Disk Susceptibility Testing of Infrequently Isolated or Fastidious Bacteria, M45.

[29] Andrews S. FastQC: A Quality Control Tool for High Throughput Sequence Data. https://www.bioinformatics.babraham.ac.uk/projects/fastqc/.

[30] Ewels P., Magnusson M., Lundin S., Käller M. (2016). MultiQC: Summarize analysis results for multiple tools and samples in a single report. Bioinformatics.

[31] Bolger A.M., Lohse M., Usadel B. (2014). Trimmomatic: A flexible trimmer for illumina sequence data. Bioinformatics.

[32] De Coster W., Rademakers R. (2023). NanoPack2: Population-scale evaluation of long-read sequencing data. Bioinformatics.

[33] Wick R.R. (2018). Filtlong: Quality filtering Tool for Long Reads. https://github.com/rrwick/Filtlong.

[34] Wick R.R., Judd L.M., Gorrie C.L., Holt K.E. (2017). Unicycler: Resolving bacterial genome assemblies from short and long sequencing reads. PLoS Comput. Biol..

[35] Gurevich A., Saveliev V., Vyahhi N., Tesler G. (2013). QUAST: Quality assessment tool for genome assemblies. Bioinformatics.

[36] Wick R.R., Schultz M.B., Zobel J., Holt K.E. (2015). Bandage: Interactive visualization of *de Novo* genome assemblies. Bioinformatics.

[37] Wood D.E., Lu J., Langmead B. (2019). Improved metagenomic analysis with Kraken 2. Genome Biol..

[38] Ondov B.D., Bergman N.H., Phillippy A.M. (2011). Interactive metagenomic visualization in a web browser. BMC Bioinform..

[39] Parks D.H., Imelfort M., Skennerton C.T., Hugenholtz P., Tyson G.W. (2015). CheckM: Assessing the quality of microbial genomes recovered from isolates, single cells, and metagenomes. Genome Res..

[40] Seemann T. (2014). Prokka: Rapid prokaryotic genome annotation. Bioinformatics.

[41] Aziz R.K., Bartels D., Best A.A., DeJongh M., Disz T., Edwards R.A., Formsma K., Gerdes S., Glass E.M., Kubal M. (2008). The RAST Server: Rapid annotations using subsystems technology. BMC Genom..

[42] Grant J.R., Enns E., Marinier E., Mandal A., Herman E.K., Chen C., Graham M., Van Domselaar G., Stothard P. (2023). Proksee: In-depth characterization and visualization of bacterial genomes. Nucleic Acids Res..

[43] Eren A.M., Kiefl E., Shaiber A., Veseli I., Miller S.E., Schechter M.S., Fink I., Pan J.N., Yousef M., Fogarty E.C. (2020). Community-led, integrated, reproducible multi-omics with Anvi’o. Nat. Microbiol..

[44] Jolley K.A., Bray J.E., Maiden M.C.J. (2018). Open-access bacterial population genomics: BIGSdb software, the PubMLST.org website and their applications. Wellcome Open Res..

[45] Feldgarden M., Brover V., Gonzalez-Escalona N., Frye J.G., Haendiges J., Haft D.H., Hoffmann M., Pettengill J.B., Prasad A.B., Tillman G.E. (2021). AMRFinderPlus and the Reference Gene Catalog facilitate examination of the genomic links among antimicrobial resistance, stress response, and virulence. Sci. Rep..

[46] Jia B., Raphenya A.R., Alcock B., Waglechner N., Guo P., Tsang K.K., Lago B.A., Dave B.M., Pereira S., Sharma A.N. (2017). CARD 2017: Expansion and model-centric curation of the comprehensive antibiotic resistance database. Nucleic Acids Res..

[47] Doster E., Lakin S.M., Dean C.J., Wolfe C., Young J.G., Boucher C., Belk K.E., Noyes N.R., Morley P.S. (2020). MEGARes 2.0: A database for classification of antimicrobial drug, biocide and metal resistance determinants in metagenomic sequence data. Nucleic Acids Res..

[48] Bortolaia V., Kaas R.S., Ruppe E., Roberts M.C., Schwarz S., Cattoir V., Philippon A., Allesoe R.L., Rebelo A.R., Florensa A.F. (2020). ResFinder 4.0 for predictions of phenotypes from genotypes. J. Antimicrob. Chemother..

[49] Liu B., Zheng D., Zhou S., Chen L., Yang J. (2022). VFDB 2022: A general classification scheme for bacterial virulence factors. Nucleic Acids Res..

[50] Schwengers O., Jelonek L., Dieckmann M.A., Beyvers S., Blom J., Goesmann A. (2021). Bakta: Rapid and standardized annotation of bacterial genomes via alignment-free sequence identification. Microb. Genom..

[51] Rabinowitz P., Zilberman B., Motro Y., Roberts M.C., Greninger A., Nesher L., Ben-Shimol S., Yagel Y., Gdalevich M., Sagi O. (2021). Whole Genome Sequence Analysis of *Brucella melitensis* Phylogeny and Virulence Factors. Microbiol. Res..

[52] Janowicz A., De Massis F., Ancora M., Cammà C., Patavino C., Battisti A., Prior K., Harmsen D., Scholz H., Zilli K. (2018). Core Genome Multilocus Sequence Typing and Single Nucleotide Polymorphism Analysis in the Epidemiology of *Brucella melitensis* Infections. J. Clin. Microbiol..

[53] Etemadi A., Moniri R., Neubauer H., Dasteh Goli Y., Alamian S. (2019). Laboratory Diagnostic Procedures for Human Brucellosis: An Overview of Existing Approaches. Jundishapur J. Microbiol..

[54] Dadar M., Alamian S., Brangsch H., Elbadawy M., Elkharsawi A.R., Neubauer H., Wareth G. (2023). Determination of Virulence-Associated Genes and Antimicrobial Resistance Profiles in *Brucella* Isolates Recovered from Humans and Animals in Iran Using NGS Technology. Pathogens.

[55] Wareth G., El-Diasty M., Abdel-Hamid N.H., Holzer K., Hamdy M.E.R., Moustafa S., Shahein M.A., Melzer F., Beyer W., Pletz M.W. (2021). Molecular characterization and antimicrobial susceptibility testing of clinical and non-clinical *Brucella melitensis* and *Brucella abortus* isolates from Egypt. One Health.

[56] Liang P.-F., Zhao Y., Zhao J.-H., Pan D.-F., Guo Z.-Q. (2020). Human distribution and spatial-temporal clustering analysis of human brucellosis in China from 2012 to 2016. Infect. Dis. Poverty.

[57] Rauthan K., Goel D., Kumar S. (2019). Annotation of a hypothetical protein (WP_002969292.1) from *Brucella abortus*. Bioinformation.

[58] Long G.S., Hider J., Duggan A.T., Klunk J., Eaton K., Karpinski E., Giuffra V., Ventura L., Prowse T.L., Fornaciari A. (2023). A 14th century CE *Brucella melitensis* genome and the recent expansion of the Western Mediterranean Clade. PLOS Pathog..

[59] Johansen T.B., Scheffer L., Jensen V.K., Bohlin J., Feruglio S.L. (2018). Whole-genome sequencing and antimicrobial resistance in *Brucella melitensis* from a Norwegian perspective. Sci. Rep..

[60] Dal T., Durmaz R., Ceylan A., Bacalan F., Karagoz A., Celebi B., Yasar E., Kilic S., Acikgoz C. (2018). Molecular Investigation of the Transmission Dynamics of Brucellosis Observed Among Children in the Province of South—East Anatolia, Turkey. Jundishapur J. Microbiol..

[61] Van T.T.H., Yidana Z., Smooker P.M., Coloe P.J. (2020). Antibiotic use in food animals worldwide, with a focus on Africa: Pluses and minuses. J. Glob. Antimicrob. Resist..

[62] Abdel-Hamid N.H., Zaffan M.R., Hamdy M.E.R., Sabry M.A., Abdelhalem M.H., Elshiekh A.Y.M., Elmonir W., Hashad M.E. (2024). Antimicrobial Susceptibility Testing and Molecular Genotyping of *Brucella melitensis* Isolates at the Human Animal Interface in Upper Egypt and Egyptian Boundaries. Egypt. J. Vet. Sci..

[63] Ötkün S., Gürbilek S.E. (2024). Whole-genome sequencing-based analysis of *Brucella* species isolated from ruminants in various regions of Türkiye. BMC Infect. Dis..

[64] Ayoub H., Kumar M.S., Mehta R., Thomas P., Dubey M., Dhanze H., Ajantha G.S., Bhilegaonkar K.N., Salih H.M., Cull C.A. (2024). Exploring genetic determinants of antimicrobial resistance in *Brucella melitensis* strains of human and animal origin from India. Front. Microbiol..

[65] Arapović J., Kompes G., Dedić K., Teskeredžić S., Ostojić M., Travar M., Tihić N., Delić J., Skočibušić S., Zekiri-Sivro M. (2022). Antimicrobial resistance profiles of Human *Brucella melitensis* isolates in three different microdilution broths: The first multicentre study in Bosnia and Herzegovina. J. Glob. Antimicrob. Resist..

[66] European Committee on Antimicrobial Susceptibility Testing (EUCAST) (2025). Breakpoint Tables for Interpretation of MICs and Zone Diameters. Version 15.0. https://www.eucast.org/fileadmin/src/media/PDFs/EUCAST_files/Breakpoint_tables/v_15.0_Breakpoint_Tables.pdf.

[67] Khan A.U., Shell W.S., Melzer F., Sayour A.E., Ramadan E.S., Elschner M.C., Moawad A.A., Roesler U., Neubauer H., El-Adawy H. (2019). Identification, Genotyping and Antimicrobial Susceptibility Testing of *Brucella* Spp. Isolated from Livestock in Egypt. Microorganisms.

[68] Morales-Estrada A., Hernández-Castro R., López-Merino A., Singh-Bedi J., Contreras-Rodríguez A. (2016). Isolation, identification, and antimicrobial susceptibility of *Brucella* spp. cultured from cows and goats manure in Mexico. Aust. J. Vet. Sci..

[69] Ravanel N., Gestin B., Maurin M. (2009). In vitro selection of fluoroquinolone resistance in *Brucella melitensis*. Int. J. Antimicrob. Agents.

[70] Valdezate S., Navarro A., Medina-Pascual M.J., Carrasco G., Saez-Nieto J.A. (2010). Molecular screening for rifampicin and fluoroquinolone Resistance in a clinical population of *Brucella melitensis*. J. Antimicrob. Chemother..

[71] Paulsen I.T., Seshadri R., Nelson K.E., Eisen J.A., Heidelberg J.F., Read T.D., Dodson R.J., Umayam L., Brinkac L.M., Beanan M.J. (2002). The *Brucella suis* genome reveals fundamental similarities between animal and plant pathogens and symbionts. Proc. Natl. Acad. Sci. USA.

[72] Ferjani A., Buijze H., Kopprio G., Köhler S., Rehaiem A., Battikh H., Ammari L., Ferjani S., Kanzari L., Zribi M. (2025). A Genomic Characterization of Clinical *Brucella melitensis* Isolates from Tunisia: Integration into the Global Population Structure. Microorganisms.

[73] Orsini M., Ianni A., Zinzula L. (2022). *Brucella ceti* and *Brucella pinnipedialis* genome characterization unveils genetic features that highlight their zoonotic potential. Microbiol. Open.

[74] Girault G., Freddi L., Jay M., Perrot L., Dremeau A., Drapeau A., Delannoy S., Fach P., Ferreira Vicente A., Mick V. (2024). Combination of in silico and molecular techniques for discrimination and virulence characterization of marine *Brucella ceti* and *Brucella pinnipedialis*. Front. Microbiol..

[75] Sibhat B., Adamu H., Asmare K., Lindahl J.F., Magnusson U., Sisay Tessema T. (2024). Detection and Molecular Diversity of *Brucella melitensis* in Pastoral Livestock in North-Eastern Ethiopia. Pathogens.

[76] Głowacka P., Żakowska D., Naylor K., Niemcewicz M., Bielawska-Drózd A. (2018). *Brucella*—Virulence Factors, Pathogenesis and Treatment. Pol. J. Microbiol..

[77] Bialer M.G., Sycz G., Muñoz González F., Ferrero M.C., Baldi P.C., Zorreguieta A. (2020). Adhesins of *Brucella*: Their Roles in the Interaction with the Host. Pathogens.

[78] Hamdy M.E.R., Zaki H.M. (2018). Detection of virulence-associated genes in *Brucella melitensis* biovar 3, the prevalent field strain in different animal species in egypt. Open Vet. J..

[79] Janowicz A., De Massis F., Zilli K., Ancora M., Tittarelli M., Sacchini F., Di Giannatale E., Sahl J.W., Foster J.T., Garofolo G. (2020). Evolutionary history and current distribution of the West Mediterranean lineage of *Brucella melitensis* in Italy. Microb. Genom..

[80] Smith J.A. (2018). *Brucella* lipopolysaccharide and pathogenicity: The core of the matter. Virulence.

[81] Mirnejad R., Jazi F.M., Mostafaei S., Sedighi M. (2017). Molecular investigation of virulence factors of *Brucella melitensis* and *Brucella abortus* strains isolated from clinical and non-clinical samples. Microb. Pathog..

[82] Ke Y., Wang Y., Li W., Chen Z. (2015). Type IV secretion system of *Brucella* spp. and its effectors. Front. Cell. Infect. Microbiol..

[83] Martínez-Núñez C., Altamirano-Silva P., Alvarado-Guillén F., Moreno E., Guzmán-Verri C., Chaves-Olarte E. (2010). The Two-Component System BvrR/BvrS Regulates the Expression of the Type IV Secretion System VirB in *Brucella abortus*. J. Bacteriol..

[84] Arellano-Reynoso B., Lapaque N., Salcedo S., Briones G., Ciocchini A.E., Ugalde R., Moreno E., Moriyón I., Gorvel J.-P. (2005). Cyclic β-1, 2-glucan is a *Brucella* virulence factor required for intracellular survival. Nat. Immunol..

[85] Hashemifar I., Yadegar A., Jazi F.M., Amirmozafari N. (2017). Molecular prevalence of putative virulence-associated genes in *Brucella melitensis* and *Brucella abortus* isolates from human and livestock specimens in Iran. Microb. Pathog..

[86] Lee J.J., Kim J.H., Kim D.G., Kim D.H., Simborio H.L., Min W.G., Rhee M.H., Lim J.H., Chang H.H., Kim S. (2014). Characterization of Betaine Aldehyde Dehydrogenase (BetB) as an Essential Virulence Factor of *Brucella abortus*. Vet. Microbiol..

[87] Oueslati I., Berriche A., Ammari L., Abdelmalek R., Kanoun F., Kilani B., Tiouiri Benaissa H. (2016). Epidemiological and clinical characteristics of neurobrucellosis case patients in Tunisia. MédecineMal. Infect..

